# The role of the microscopic world: Exploring the role and potential of intratumoral microbiota in cancer immunotherapy

**DOI:** 10.1097/MD.0000000000038078

**Published:** 2024-05-17

**Authors:** Liqiang Zhang, Liang Yu

**Affiliations:** a Department of Oncology, Weifang Hospital of Traditional Chinese Medicine, Weifang City, Shandong Province, China; b Department of Cardiac Surgery, Weifang Hospital of Traditional Chinese Medicine, Weifang City, Shandong Province, China.

**Keywords:** antitumor therapy, intratumoral microbiome, tumor immunology

## Abstract

Microorganisms, including bacteria, viruses, and fungi, coexist in the human body, forming a symbiotic microbiota that plays a vital role in human health and disease. Intratumoral microbial components have been discovered in various tumor tissues and are closely linked to the occurrence, progression, and treatment results of cancer. The intratumoral microbiota can enhance antitumor immunity through mechanisms such as activating the stimulator of interferon genes signaling pathway, stimulating T and NK cells, promoting the formation of TLS, and facilitating antigen presentation. Conversely, the intratumoral microbiota might suppress antitumor immune responses by increasing reactive oxygen species levels, creating an anti-inflammatory environment, inducing T cell inactivation, and enhancing immune suppression, thereby promoting cancer progression. The impact of intratumoral microbiota on antitumor immunity varies based on microbial composition, interactions with cancer cells, and the cancer’s current state. A deep understanding of the complex interactions between intratumoral microbiota and antitumor immunity holds the potential to bring new therapeutic strategies and targets to cancer immunotherapy.

## 1. Introduction

Various microorganisms, such as bacteria, viruses, and fungi, inhabit the human body, forming a symbiotic microbial community that is crucial for human health.^[1,2]^ Many studies of the human microbiome indicate that the microbiota differs between healthy and diseased individuals. In particular, the microbiota has a close relationship with cancer; it affects carcinogenesis in the human body.^[3]^ For instance, in colorectal cancer (CRC), *Fusobacterium nucleatum* (Fn) can adhere, invade, and initiate oncogenic and inflammatory responses through their distinctive FadA adhesin.^[4]^ Similarly, Fn can stimulate tumor cells to produce specific exosomes that promote CRC metastasis.^[5]^ Garrett et al^[6]^ reported 3 ways in which the microbiota may lead to tumor progression and development: (1) changing the balance of cell proliferation and apoptosis, (2) reprogramming the immune system and responses, and (3) affecting the metabolism of host-secreted factors, foods, and drugs.

Many studies have shown that the gut microbiota is essential for the regulation of host immune responses. However, the intratumoral microbiota may also play a key role in shaping the local immune responses of the tumor microenvironment, which further affects tumor progression. The intratumoral microbiota play different roles in antitumor immunity: by either enhancing or decreasing antitumor immune responses and inducing different immunotherapy efficacies and outcomes.^[7,8]^

This article provides an overview of the origin of intratumoral microbial communities, their impact on anti-tumor immunity, and their role in cancer treatment, particularly in immunotherapy. These discoveries have the potential to introduce novel therapeutic approaches and targets for tumor immunotherapy, offering new directions and possibilities for cancer treatment.

## 2. Source of intratumor microbiota

Recent studies indicate that the sources of intratumoral microbes can be categorized into 3 types: 1. mucosal sites, 2. normal adjacent tissues (NATs), and 3. hematogenous spread. The hypoxic and nutrient-rich tumor microenvironment, coupled with immune suppression, creates favorable conditions for microbial colonization.

Intratumoral microbes are commonly present in cancers related to mucosal sites, such as lung cancer, esophageal cancer, pancreatic cancer, cervical cancer, and colorectal cancer.^[3,9]^ The breakdown of the mucosal barrier in the body due to tumor development or other reasons provides an opportunity for these intratumoral microbes to invade, allowing them to colonize within the tumor microenvironment (TME). Tjalsma proposed that enterotoxigenic *Bacteroides fragilis (B fragilis*) and *Helicobacter pylori (H pylori*) may induce tumors, creating conditions for other microbes to enter the TME. This implies that certain microbes have specific roles in enabling other microbes to infiltrate the TME.^[10]^ Several studies have emphasized the presence of intratumoral bacteria in pancreatic ductal adenocarcinoma, indicating their potential origin from the gut and possible spread through the pancreatic duct.^[11–13]^ Further research is crucial to reveal the exact mechanisms through which microbes enter the TME from mucosal organs. Understanding these mechanisms is essential to enhance the effectiveness of bacterial transplantation into mucosal organs, especially techniques like fecal microbiota transplantation.

The conventional belief was that intratumoral microbes originate solely from mucosal sites. However, the presence of specific microbes, such as in breast cancer, challenges this notion in non-mucosal tumors. Recent studies have indicated that bacteria within tumors might originate from NATs. Observations reveal significant similarities in bacterial composition between tumor tissues and NATs.^[8]^ The discovery of microbes inside tumors, including those from non-mucosal sites, raises questions about their diverse origins. This suggests that specific tumor microenvironments, such as immune suppression and hypoxia, might have potential influences. The exact origins of microbes in NATs remain uncertain, and it is unclear whether they significantly contribute to intratumoral microbiota. The complex interactions between intratumoral microbes, normal adjacent tissues, and the tumor microenvironment underscore the need for extensive research to elucidate the intricate origins and dynamic processes of these microbes within cancer tissues.

The circulatory system might be a potential source of intratumoral microbes, particularly with hematogenous dissemination being a key mechanism for their colonization within the tumor microenvironment. *F nucleatum* can CRC through hematogenous dissemination, facilitated by the interaction between Fap2 lectin and Gal-GalNAc expressed in CRC.^[14]^ Similarly, *F nucleatum* can invade breast cancer through hematogenous dissemination, with Fap2 lectin aiding in this colonization process.^[15]^ Microbes from the oral cavity, intestines, and other potential sites can be transported via the bloodstream to tumor locations, entering tumors through damaged blood vessels, thereby emphasizing the diverse origins and pathways of intratumoral microbes.

The origins and mechanisms of intratumoral microbes are gradually being unveiled, allowing for a deeper understanding of the dynamics and behavior of the intratumoral microbiota in cancer tissues. Comparing the composition of intratumoral microbiota with microbiota from other body sites can identify key microbes associated with different tumors, offering valuable insights for cancer prevention strategies. Exploring the molecular mechanisms of how microbes invade the TMEs remains a fascinating and crucial research area. Despite our current limited understanding, in-depth research into these mechanisms may reveal key pathways of microbial interactions within cancer tissues.

## 3. Signaling pathways involved in the influence of intratumoral microbiota immune response

### 3.1. TLR signaling

The Toll-like receptor (TLR) signaling pathway plays a crucial role in the complex influence of microbes on tumor development. *F nucleatum* activates NF-κB, ERK, and AKT pathways in colon cancer cells via TLR4 receptors, increasing inflammatory factors and metabolites and driving tumor progression.^[6,16,17]^ Lipopolysaccharides are essential components of the outer membrane of Gram-negative bacteria. By recognizing lipopolysaccharides, TLR4 activates the NF-κB signaling pathway, leading to the release of inflammatory cytokines (IL-1β, IL-6, IL-8, TNF-α).^[18,19]^

Microbial binding to TLRs on immune cells initiates the immunosuppressive inflammation. Studies have shown that symbiotic bacteria activate MYD88 in myeloid cells, leading to the secretion of IL-23 and IL-1β, stimulating γδ T cells to produce IL-17, triggering local inflammation, and promoting the progression of lung cancer.^[20]^ In pancreatic adenocarcinoma, microbes binding to TLRs on monocytes transform them into tumor-associated macrophages (TAMs), increase myeloid-derived suppressor cells (MDSCs), and inhibit the differentiation of CD4 T cells into Th1 cells, forming a suppressive tumor microenvironment.^[13]^

### 3.2. NF-κB signaling

The activation of NF-κB by microbes through the β-catenin and Toll-like receptor pathways creates a highly inflammatory microenvironment within the tumor.^[21]^ This chronic inflammation, characterized by the release of inflammatory factors and the recruitment of immune cells, provides a conducive milieu for tumor progression. *F nucleatum* can activate NF-κB-driven inflammatory responses to promote tumor progression.^[22,23]^ Additionally, the enterotoxin (Bft) also activates the MAPK pathway, enhances NF-κB signaling, and triggers mucosal inflammation response in intestinal epithelial cells.^[24–27]^

### 3.3. STING signaling

STING (stimulator of interferon genes) is a cytoplasmic DNA-sensing protein activated by cyclic dinucleotides, leading to the expression of interferon-β and pro-inflammatory genes.^[28]^ STING is expressed in various cells, including macrophages and dendritic cells (DCs), activating the cGAS/STING signaling pathway, polarizing M1 macrophages, and exerting potent anti-tumor effects.^[29–32]^ Certain bacteria, such as *B fragilis*, produce STING agonists, inducing polarization of anti-tumor macrophages, promoting NK cell activation, and enhancing interaction with DCs.^[33]^ Lactobacillus strains induce the production of cGAS/STING-dependent interferon-I, activating DCs and recruiting tumor-specific CD8 T cells into the tumor microenvironment.^[34,35]^

### 3.4. ROS signaling

Microorganisms induce an increase in reactive oxygen species (ROS) in myeloid cells, leading to mitochondrial ROS, DNA damage, reduced NAD levels, and subsequent aging of M1 macrophages, thereby affecting their functions and behavior.^[36,37]^ MDSCs from myeloid lineage produce peroxynitrite in a ROS-dependent manner, suppressing T cell function and weakening the immune response against tumors.^[38]^

### 3.5. β-catenin signaling

Microorganisms such as *H pylori, Salmonella typhi (S typhi*), *F nucleatum*, and *B fragilis* activate the β-catenin signaling pathway through direct or E-cadherin-mediated mechanisms.^[4,18,39–41]^ Salmonella effector protein AvrA upregulates the expression of Wnt and TCF/Lef1, promoting posttranslational modification of β-catenin and activating downstream target genes like c-Myc and cyclin D1.^[42–44]^ *F nucleatum*’s FadA, by phosphorylating E-cadherin, triggers a series of events, including the migration of β-catenin into the nucleus, activation of transcription factors (TCF, NF-κB), and enhancement of oncogenes (Myc) and cell cycle protein D1.^[23]^ The Bft protein in *B fragilis* triggers the cleavage of E-cadherin, promoting TCF-dependent nuclear translocation of β-catenin and increasing the expression of inflammatory genes.^[4,41,45]^

## 4. The impact of intratumoral microbiota on anti-tumor immunity

Studies have shown that intestinal microbiota can enhance the effectiveness of immunotherapy, particularly through checkpoint inhibition, by regulating anti-tumor immune responses.^[36,46–50]^ Interestingly, intestinal microbiota can also suppress tumor immune responses, indicating its diverse impact on anti-tumor immunity.^[51–53]^ Similarly, intratumoral microbiota are believed to shape the tumor immune microenvironment, exerting unique roles in enhancing anti-tumor immunity and immunotherapy effectiveness, or dampening anti-tumor immune responses, thereby influencing cancer progression.

### 4.1. Enhancing antitumor immune response

Intratumoral microbiota, activate the STING signaling pathway, stimulating immune responses, including T cells, and NK cells, fostering tertiary lymphoid structures (TLS), and enhancing anti-tumor immunity through specific antigen presentation.^[54,55]^

### 4.2. STING signaling activation

Intratumoral microbiota can enhance anti-tumor immunity by activating the STING signaling pathway. As part of the intratumoral microbiota, Bifidobacterium activates DCs through the STING signaling pathway. The systemic and local administration of *Bifidobacterium* can enhance cancer treatment by reshaping the tumor microenvironment, promoting DCs’ cross-priming, and improving T cell responses through the STING-dependent mechanism.^[34]^ The STING agonist produced by *Akkermansia muciniphila* enhances the efficacy of immune checkpoint inhibitors by stimulating the production of IFN-I, inducing macrophage reprogramming, and enhancing cross-communication between NK cells and DCs.^[33]^

### 4.3. T and NK cell activation

The presence of specific microbes (*Saccharopolyspora, Pseudoxanthomonas*, and *Streptomyces*) within pancreatic adenocarcinoma tissues enhances the activity of CD8 T cells and granzyme B cells, leading to a potent anti-tumor immune response and improved overall survival.^[55]^ The discovery of Clostridiales-produced trimethylamine N-oxide (TMAO) triggering PERK-mediated endoplasmic reticulum stress enhances anti-tumor immunity and improves immunotherapy efficacy in triple-negative breast cancer (TNBC).^[56]^ Intratumoral microbiota, including members of the order Clostridiales, EBV and HBV, trigger the generation of chemotactic factors, enhance the infiltration of CD8 T cells into tumor tissues, and increase the survival rates of melanoma patients.^[57–61]^ High-salt diet-induced enrichment of intratumoral Bifidobacterium enhances the function of NK cells and promotes melanoma reversal by elevating the levels of metabolic product glucosylceramides.^[62]^ In melanoma, *Eubacterium* rectale can manipulate l-serine levels through the Fos/Fosl pathway to promote NK cell activation and enhance NK cell function.^[63]^

### 4.4. TLS production

The intratumoral microbiota plays a crucial role in promoting the maturation of TLS. Mature TLS facilitate the infiltration and activation of lymphocytes within tumor tissues, creating a favorable microenvironment for anti-tumor immune responses. *H pylori* inside tumors triggers anti-tumor immune responses relying on T follicular helper cells (Tfh) and B cells.^[64]^ These responses support the formation of TLS in the surrounding tumor area, thereby inhibiting the growth of colon cancer.

### 4.5. Intratumoral microbiota-derived antigen presenting

Bacteria inside tumors are present both in tumor cells and immune cells. Experimental evidence demonstrates that exposure to bacterial peptides increases the secretion of IFN-γ by melanoma-infiltrating lymphocytes, triggering an immune response in T cells, making it a potential target to attack tumor cells.^[8,54]^

### 4.6. Decreasing antitumor immune response

The bacterial community within tumors plays a dual role in cancer progression. On one hand, they can enhance anti-tumor immunity by triggering immune cell responses, stimulating anti-tumor immune reactions, and thereby aiding in resisting the growth and spread of cancer cells. At the same time, they can also promote cancer progression by increasing ROS levels, promoting an inflammatory environment, inducing T cell inactivation, and immune suppression mechanisms, providing favorable conditions for the survival and proliferation of cancer cells.

### 4.7. Upregulation of ROS

The bacterial community within tumors, such as *B fragilis*, produces reactive oxygen species (ROS), triggering inflammation and immune suppression mechanisms, modulating immune responses, and thereby driving the development of colorectal cancer.^[3,6]^ *Fusobacterium* is primarily distributed in the gastrointestinal tract, promoting the occurrence of colorectal cancer through ROS-mediated local inflammation.^[51,65]^

### 4.8. Promote an inflammatory environment

The microbial community within tumor tissues modulates the tumor microenvironment to promote cancer progression. Bacteria within the tumor secrete IL-17, promoting the growth of colorectal cancer by increasing B cell infiltration in the tumor tissue.^[66]^ Symbiotic bacteria stimulate γδ T cells to produce IL-17, enhancing local inflammation in lung cancer and driving tumor development.^[20]^ The fungi in pancreatic cancer enhance the secretion of IL-33, recruiting Th2 cells and ILC2 into the tumor tissue, facilitating tumor progression.^[67]^

### 4.9. T cell inactivation

Recent studies indicate that the microbial community within tumors may lead to a decrease in the number and functionality of T cells in the tumor microenvironment. In breast cancer tissue, the presence of *F nucleatum* significantly reduces T cell infiltration, diminishing their ability to combat cancer cells, thus promoting the growth and metastasis of breast cancer.^[15]^ The number of bacteria within tumors shows a negative correlation with T cell infiltration.^[68,69]^ In gastric cancer tissue, the presence of *Methylobacterium* impairs the function of CD8 tissue-resident memory cells, accelerating tumor development.^[70]^

### 4.10. Immunosuppression

Specific microbes such as *Nevskia ramosa* and *Staphylococcus aureus*, as well as viruses like HBV and HCV, promote the development of prostate cancer and liver cancer by modulating regulatory T cells and manipulating immune-related genes.^[71–73]^ Antibiotic treatment in mice reduces the microbial population within tumors, which is associated with decreased activation of Tregs, T cells, and NK cells, inhibiting melanoma metastasis.^[74]^ The microbiota in pancreatic ductal adenocarcinoma regulates TAMs through TLR signaling, creating an immunosuppressive tumor microenvironment.^[13]^ Clearing the intra-tumoral microbiota can reshape the tumor microenvironment, increasing the number of M1-type macrophages, activating CD8 T cells, and reducing MDSCs. Additionally, it synergistically enhances the efficacy of immunotherapy by upregulating the expression of programmed death protein 1 (PD-1).^[13]^ Symbiotic fungi promote the generation of tumor-promoting macrophages by binding to Dectin-1, reducing the number of T cells, thereby suppressing the immune response.^[75]^

### 4.11. Condition-dependent effects

The impact of intra-tumoral microbiota on anti-tumor immunity varies depending on specific tumor conditions. The presence of *F nucleatum* within colorectal cancer tissues exhibits a dual impact, negatively correlated with tumor-infiltrating lymphocytes in microsatellite instability-high tumors, suggesting immune suppression, while showing a positive correlation in non-microsatellite instability-high cases, indicating potential immune activation. The alliance between MSI-high colorectal tumors and *F nucleatum* creates an immunosuppressive microenvironment dominated by M2-type macrophages, fostering tumor promotion and immune evasion.^[76]^ This indicates the existence of intricate interactions between intra-tumoral microbiota, MSI status, and anti-tumor immunity.^[77]^

Intra-tumoral microbiota may play both positive and inhibitory roles in regulating anti-tumor immune responses. Different types of tumor tissues interact with various intra-tumoral microbiota in diverse ways, thereby influencing immune responses. The key factors determining how intra-tumoral microbiota influence anti-tumor immunity are still unclear. Understanding the complexity of immune responses mediated by microbial communities is crucial for optimizing immunotherapy strategies. The pancreas, connected to the digestive tract, has become a focal area of research. Microbes from external sources and the gut may colonize pancreatic tissues and play a crucial role in modulating anti-tumor immune responses. Understanding the microbial communities is vital for enhancing the effectiveness of immunotherapy. Future research should focus on the intricate interactions within intra-tumoral microbial communities and their impact on cancer immunotherapy.

## 5. The application of microbiota in cancer treatment

The intratumoral microbiota is actively being applied in cancer treatment, marking a significant change in treatment strategies. By employing targeted engineering methods to enhance the anti-tumor effects of microbiota, their efficacy can be maximized. The intratumoral microbiota can stimulate innate and adaptive immunity, thereby enhancing the body’s anti-tumor immune response.^[78–86]^ Several microbiota and related formulations have already received FDA approval, validating their reliability and safety in cancer treatment.^[87–89]^

### 5.1. Vectors for cancer therapy

The microbiota possess a unique ability to selectively grow within tumor tissues and can deliver anticancer drugs, making them valuable in targeted therapy. Utilizing engineered microbiota designed to colonize specific tumor tissues, leveraging their natural properties, enhances the efficacy of cancer treatment.^[90–92]^

Utilizing engineered bacterial vectors to express cytotoxic drugs has brought about innovations in cancer treatment.^[93]^ These vectors enable targeted delivery and production of potent anti-cancer drugs within the tumor microenvironment. These engineered bacteria can serve as independent therapeutic methods or be used in combination with other anti-cancer strategies.^[93]^ In mouse models, treatment with attenuated strains of *Listeria* significantly reduced tumor metastasis.^[94]^ Engineered *Escherichia coli* systems have shown promising results in inducing tumor regression.^[95]^

The microbiota plays a crucial role in cancer treatment, particularly in gene delivery. Researchers have developed a groundbreaking DNA delivery system using *Listeria*, enabling precise in vivo gene delivery and targeted cancer therapy.^[96]^ Scientists have developed engineered *Salmonella* strains armed with Fas ligand, enabling precise anti-tumor responses by inducing apoptosis in cancer cells.^[97]^ Through bacterial-mediated cytotoxic Cp53 peptide expression, bacteria self-lyse, releasing Cp53, ultimately killing tumor cells.^[98]^ These innovative approaches showcase the promising results achievable in anti-tumor responses and highlight the potential of microbiota as therapeutic vectors.

### 5.2. Regulating immune responses against tumors

The microbiota not only has the ability to deliver drugs or genes but also can stimulate the body’s innate and adaptive immunity, demonstrating its diverse anti-tumor effects.^[79,80,82,93]^We summarized the microbiota regulating immune responses for cancer treatment (Table 1).

**Table 1:** Regulation of the immune responses by the microbiota used for cancer therapy.

Classification	Therapeutic strategy	Microbiota type	Tumor type	Effect	Ref
Engineered microbiota expressing tumor antigens	Tumor vaccine	Listeria and Salmonella	Solid tumor	Delivery of tumor antigens to the immune system	^[99]^ ^,[100]^
	Express immunodominant T cell antigens to infect tumor cells	Tetanus toxoid, poliovirus, or measles virus	Solid tumor	Induction of memory T cell responses	^[93]^
Engineered microbiota expressing immunoregulatory factors	Bacteria-mediated DNA delivery	Nonpathogenic bacteria	Ovarian and colon cancer	Recruitment of more phagocytic cells, enhancement of inflammatory responses	^[90]^
	Salomonella expressing β-galactosidase	S. typhimurium	NA	Induction of substantially stronger immune responses	^[101]^
	Induction of substantially stronger immune responses	*S. typhimurium*	Colon cancer	Reduction of tumor progression, polarization of TAMs to M1-like macrophages, increased proinflammatory cytokine production	^[91]^
	Salmonella expressing IL-2	Salmonella	Colon cancer with liver metastasis	Enhancement of antitumor immunity in a NK and CD8 T cell-depended way	^[102],[103]^
	Salmonella expressing LIGHT	Salmonella	Breast, colon, and lung cancer	Induction of CD4 and CD8 T cell response	^[104]^
	Listeria with α-galactosylceramide	Listeria	Breast cancer	Stimulation of NKT cells, inhibition of tumor progression	^[105]^
Microbiota combined with checkpoint inhibitors	Bifidobacterium + anti-PD-L1 antibody	Bifidobacterium	Breast cancer	Inhibition of tumor growth, enhancement of cancer immunotherapy	^[106]^
	Talimogene laherparepvec (Tvec) + pembrolizumab	Tvec	Advanced melanoma	Objective response rate was 62%, reprogramming of immunogenic microenvironment	^[107]^
	Microbiota engineered with secreting checkpoint inhibitors	*C novyi*-NT and *C sporogenes*	Solid cancer	Immune activation of T cells	^[108]^

Probiotics play a crucial role in regulating the gut microbiota and enhance the body’s natural defense against tumors by influencing inflammatory responses.^[109]^ Through oral administration, probiotic DTA81 can inhibit the development of colon cancer.^[110]^

Studies have observed that non-engineered microbiota, including nonpathogenic strains of *Escherichia coli*, can enhance anti-tumor immunity. The *E. coli* strain MG1655 targets tumor cells by producing high levels of TNF-α, paving the way for new directions in cancer treatment.^[111]^ Researchers have discovered that immunogenic gut bacteria activate Tfh cells,^[64]^ promoting anti-tumor immune responses in colorectal cancer tissue.

Engineered bacterial strains expressing tumor antigens and immune-related factors activate specific immune responses, thereby enhancing anti-tumor immunity. Studies have shown that live bacterial populations can serve as carriers for tumor antigens in cancer vaccine administration.^[99,100,112]^ Scientists have engineered bacterial strains expressing immunodominant T-cell antigens, infiltrating tumor cells to activate specific and enduring memory T-cell responses.^[93]^ Memory T cells recognize antigens in tumor cells, facilitating the destruction of infected tumor cells and potentially triggering anti-tumor immune responses in neighboring uninfected cells. This approach holds promise for innovative cancer immunotherapy and vaccine development.

Engineered microbial communities induce and activate immune responses in cancer therapy by expressing immune modulating factors. Bacteria-mediated DNA delivery enhances immune responses and anti-tumor effects.^[90]^ *Salmonella* can serve as an effective delivery vector for immune modulating factors such as β-galactosidase, enhancing immune responses.^[101]^ Researchers engineered *Salmonella typhimurium* to produce Vibrio vulnificus flagellin B, a highly immunogenic molecule. This factor strengthens the immune response by activating TLR5 and ligand signaling pathways, polarizing TAMs into M1-type macrophages, and enhancing the secretion of IL-1β and TNF-α, thereby slowing down tumor progression.^[91]^ The *S typhimurium* strains expressing IL-2 can enhance anti-tumor immune response by activating NK cells and CD8 T cells.^[102,103]^ Engineering of *S typhimurium* strains expressing lymphotoxin affects the immune response of CD4 and CD8 T cells.^[104]^ *Listeria* monocytogenes carrying α-galactosylceramide can stimulate NKT cells, inhibit tumor growth, and prolong patients’ survival.^[105]^ These pioneering studies demonstrate the potential of engineered bacteria as weapons in cancer treatment, harnessing the diversity of the immune system components. By activating NK cells, CD8 T cells, CD4 T cells, and NKT cells, researchers are paving the way for more targeted and effective cancer therapies.

Combining the microbiota with checkpoint inhibitors enhances the effectiveness of cancer immunotherapy.^[107,113,114]^ Through oral administration, *Lactobacillus*, a beneficial gut bacterium, joins forces with anti-PD-L1 antibodies, resulting in a remarkable alliance that significantly impedes tumor growth.^[106]^ The combination therapy of *Talimogene Laherparepvec*, an oncolytic herpes virus, with Pembrolizumab, a PD-1 checkpoint inhibitor, has achieved a remarkable 62% objective response rate in advanced melanoma patients.^[107]^ Infection of the tumor microbiota restructures the immunogenic microenvironment, enhancing the tumor’s sensitivity to immune checkpoint inhibitors. Engineered microbiota, modified to produce anti-PD-1 antibodies, represent a revolutionary approach in cancer immunotherapy, providing sustained, localized, and targeted treatment by activating T cells and disrupting immune evasion tactics within the tumor microenvironment.^[108]^ This innovative treatment approach holds tremendous potential in the field of cancer therapy. Scientists are pioneering new avenues by harnessing the complex interactions between the tumor microbiota, the immunogenic microenvironment, and immune checkpoint inhibitors, making tumors more responsive and thereby improving the effectiveness of immunotherapy.

## 6. Conclusions

In summary, the composition of the tumor microbiota plays a crucial role in shaping the tumor immune microenvironment. It can either promote inflammatory responses or inhibit anti-tumor effects. The impact of the tumor’s internal microbiota on anti-tumor immunity depends on various factors, including microbiota composition, interactions with cancer cells, and specific cancer conditions.

Although recent research on the tumor microbiota has made progress, there are still limitations in understanding its mechanisms of impact on anti-tumor immunity and treatment outcomes, which hampers its clinical applications. It is essential to extensively validate these interactions’ complexity through preclinical models and clinical trials. Future treatments may involve interventions targeting the microbiota or combining microbiota-related approaches with immunotherapy to enhance the effectiveness of cancer treatments.

## Acknowledgments

We acknowledge the Department of Oncology of Weifang Hospital of Traditional Chinese Medicine.

## Author contributions

**Writing – original draft:** Liqiang Zhang.

**Writing – review & editing:** Liang Yu.
